# 

**Published:** 1894-06-02

**Authors:** 


### CONTENTS.

**Medical Aid Associations** - 177

**Around the Hospitals** - 178

**On Modern Progress in Ophthalmic Medicine and Surgery.** By ROBERT BRUDENELL CARTER, F.R.C.S. - 179

**Medical Progress and Hospital Clinics—**

The Urine in Typhoid Fever - 185

Exophthalmic Goitre and Its Relation to Diseases of the Thyroid Gland (*continued*) - 186

PROGRESS IN SURGERY.—Surgery of the Œsophagus and Stomach 187

Surgery of the Intestines - 188

PROGRESS IN GYNÆCOLOGY.—Myoma Uteri and Its Treatment - 190

NEW DRUGS AND PREPARATIONS - 192

**Construction Notes—Scraps and Gleanings** - 197

**The First Century of English Medical Literature.** IV. —The Library of the Physician - 180

**Typhus Fever and Difficulties in Diagnosis** - 181

**The Field of Science** - 182

**Annotations.**—The British Medical Benevolent Fund—Some Justice for Doctors—Political Insanity - 183

**Notes and News** - 184

**The Institutional Workshop—**

THE GENERAL MEDICAL COUNCIL - 193

THE BIRMINGHAM HOSPITAL SATURDAY FUND - 194

PRACTICAL DEPARTMENTS - 194

HOSPITAL FESTIVALS AND MEETINGS - 195

EDITOR'S LETTER-BOX - 196

### EXTRA SUPPLEMENT.—THE NURSING MIRROR.

**News from the Nursing World.**—The Queen and the Nurses —Not Good Enough for the Poor—Drained on the Pavements —An Incompetent Caretaker—Nurses and Small-pox—Newlyn Nursing Association—A Nursing Home at Leyton—Hospital Saturday at Dudley—Cottage Nurses—Working with the Nurse—District Nursing in Aberdeen—Nursing and Kindliness in Belfast—Nurses' Sitting-rooms—Short Items - lxxxi
**On General Nursing.** XIV.—Rheumatism - lxxxiii
**Duty versus Danger** - lxxxiii
**Nursing in New South Wales** - lxxxiii
**The Mechanism of Movement.** I.—The Composition of Bone - lxxxiv
**Appointments** - lxxxiv
**On the Nursing of Enteric Fever in Children** - lxxxv
**Notes and Queries** - lxxxv
**English Nurses in Brazil—Nursing in Switzerland** - lxxxvi
**Certified Asylum Attendants** - lxxxvi
**District Nurses** - lxxxvii
**Practical Suggestions.**—A Ten Centime Piece - lxxxviii
**Presentations** - lxxxviii
**The Muse's Looking-Glass** - lxxxix
**Where to Go** - lxxxix
**Roden Noel** - xc
**Everybody's Opinion—Wants and Workers** - xc

